# Proceedings of a Meeting of Associated Alumni of Dental Colleges

**Published:** 1853-04

**Authors:** C. R. Coffin, D. P. Gregg

**Affiliations:** Prest. P. T.; Secretary P. T.


### ALUMNI OF DENTAL COLLEGES.

*Proceedings of a Meeting of Associated Alumni of Dental Colleges.*—March 23d, 1853.—At a meeting of the members of the dental profession held at the Baltimore College for the purpose of taking into consideration the propriety of organizing a society for the purpose of elevating the dental art, on motion of Dr. Colburn, Dr. Coffin was called to the chair, and Dr. Gregg was appointed Secretary.

Drs. Dwinelle, Thackston and Ballard being severally called upon, respectively advocated the necessity of forming a society at the present time to sustain and enhance the position of the dental profession. A further exposition of the objects of the meeting were set forth by Prof. Blandy and Dr. Colburn. On motion of Dr. Ballard a committee of three were appointed by the chair to draft resolutions, constitution and by-laws. The committee as appointed consisted of Prof. Blandy, Drs. Dwinelle and Thackston. On motion of Dr. Gregg, Dr. Ballard was added to that committee.

After a variety of remarks by different gentlemen, the meeting adjourned to convene to-morrow at nine o'clock.

March 24th, 1853.

The meeting was called to order by the chairman, according to adjournment, when the proceedings of yesterday were read and approved. The committee being ready, made their report. Remarks were then made by Dr. Ballard, suggesting a name for the society. On motion of Dr. Gregg, the constitution, as reported by the committee was adopted as a whole. On motion the blank in Art. —— be filled by inserting the name "Associated Alumni of American Dental Colleges."

On motion of Dr. Ballard a committee of three were appointed to confer with the committee of revision of the Constitution of the "American Society of Dental Surgeons" to secure their co-operation to effect a more perfect organization. Drs. Gregg, Wadsworth and Ballard appointed on that committee. On motion the meeting adjourned to meet again at three o'clock.

March 24th, 1853, 3 o'clock.

The meeting met pursuant to adjournment, when the proceedings of the last meeting were read and approved. The following officers were then elected for the ensuing year: Prof. Blandy, President; Dr. Thackston, Vice-President; Dr. Gregg, Treasurer; Dr. M. French, Cor. Sec'y.; and Dr. Wayland, Rec. Sec'y. The following committees were then selected: On dental practice, Dr. Wadsworth; on mechanical dentistry, M. D. French; Drs. Austin, Thackston and Arthur, a committee to select subjects for scientific investigation. Prof. Townsend was unanimously selected to deliver the annual address, and Prof. C. A. Harris, his alternate. Adjourned to meet again on the 26th at 8 o'clock.

March 26th, 1853.

The chairman called the meeting to order, proceedings of former meetings read and approved. Prof. C. A. Harris entertained the meeting with an interesting address on the objects and influences of this society. On motion of Prof. Blandy, a reconsideration of the motion to appoint a committee of conference was carried, and the same committee continued with instructions to address a circular to the Alumni of the different dental colleges, and give a synopsis of the objects and constitution of this society. On motion the committee appointed to draft constitution and by-laws be continued with instructions to report at our next meeting any emendations necessary for our government. On motion of Prof. C. A. Harris, this committee was instructed to incorporate into the by-laws a provision to enable this society to make such donations or loans to the necessities of any of its members as it may think proper.

Professors C. A. Harris and Blandy then made a present to the society of a full copy of the American Journal of Dental Science, as well as a copy of Harris' Principles and Practice of Dental Surgery. Professor Blandy then gave the society sundry volumes of the Medical Journal. On motion of Dr. Gregg, seconded by Dr. Dwinelle, the thanks of this society were tendered to Profs. C. A. Harris and Blandy for their donations, carried unanimously. On motion of Prof. C. A. Harris, Dr. Dwinelle was appointed to read a paper at our next meeting on microscopic dental anatomy. Dr. Thackston was appointed to read an essay on dental education, Prof. Blandy on obturators, Prof. C. A. Harris on diseases of the dental pulp, Dr. Gregg on professional etiquette. On motion of Dr. Thackston, seconded by Dr. Dwinelle, Drs. Arthur and Flagg, (of Philadelphia,) be requested to prepare an essay on dental science. On motion of Dr. Harris, Dr. Maynard be requested to make a suitable drawing for a seal for this society. On motion of Dr. Gregg, the American Journal of Dental Science, Dental News Letter, Dental Recorder, and Dental Register of the West, be requested to publish the proceedings of our meetings—carried.

On motion, Dr. Gregg, gave a few words at parting, followed by Dr. Dwinelle, Prof. Townsend and Prof. C. A. Harris. On motion, the society adjourned to meet the day before the next annual commencement of the Baltimore College in this place.

C. R. COFFIN, *Prest. P. T.*

D. P. GREGG, *Secretary P. T.*

